# Clinical Features and Treatment Progress of Invasive Mucormycosis in Patients with Hematological Malignancies

**DOI:** 10.3390/jof9050592

**Published:** 2023-05-19

**Authors:** Nuobing Yang, Lining Zhang, Sizhou Feng

**Affiliations:** 1 State Key Laboratory of Experimental Hematology, National Clinical Research Center for Blood Diseases, Haihe Laboratory of Cell Ecosystem, Institute of Hematology & Blood Diseases Hospital, Chinese Academy of Medical Sciences & Peking Union Medical College, Tianjin 300020, China; yangnuobing@ihcams.ac.cn (N.Y.); zhanglining@ihcams.ac.cn (L.Z.); 2 Tianjin Institutes of Health Science, Tianjin 301600, China

**Keywords:** invasive mucormycosis, hematological malignancies, high risk factors, clinical manifestations, treatment

## Abstract

The incidence rate of invasive mucormycosis (IM) in patients with hematological malignancies (HMs) is increasing year by year, ranging from 0.07% to 4.29%, and the mortality rate is mostly higher than 50%. With the ongoing pandemic of COVID-19, COVID-19-associated mucormycosis (CAM) also became a global health threat. Patients with high risk factors such as active HMs, relapsed/refractory leukemia, prolonged neutropenia may still develop breakthrough mucormycosis (BT-MCR) even under the prophylaxis of *Mucorales*-active antifungals, and such patients often have higher mortality. *Rhizopus* spp. is the most common genus associated with IM, followed by *Mucor* spp. and *Lichtheimia* spp. Pulmonary mucormycosis (PM) is the most common form of IM in patients with HMs, followed by rhino-orbital-cerebral mucormycosis (ROCM) and disseminated mucormycosis. The prognosis of IM patients with neutrophil recovery, localized IM and receiving early combined medical–surgical therapy is usually better. As for management of the disease, risk factors should be eliminated firstly. Liposome amphotericin B (L-AmB) combined with surgery is the initial treatment scheme of IM. Those who are intolerant to L-AmB can choose intravenous formulations or tablets of isavuconazole or posaconazole. Patients who are refractory to monotherapy can turn to combined antifungals therapy.

## 1. Introduction

In recent years, the incidence rate of invasive mucormycosis (IM) in patients with hematological malignancies (HMs) increased, making it the most common disease among non-*Aspergillus* invasive mold infections (NAIMIs) [1]. Acute leukemia, allogeneic hematopoietic stem cell transplantation (allo-HSCT), chronic severe neutropenia, use of immunosuppressants, complicated with diabetes mellitus, iron overload, and use of deferoxamine to reduce serum iron are all high risk factors for HMs patients to be infected by *Mucorales*. Although IM is relatively rare in patients with HMs, its mortality rate is over 50% [2,3], which deserves clinical attention. In addition, the ongoing COVID-19 pandemic leads to immune dysregulation and increased use of steroids, thus making COVID-19 patients more susceptible to *Mucorales* and contributing to the surge of COVID-19-associated mucormycosis (CAM) cases. In order to improve the treatment status of this population and reduce its mortality, this article reviews the clinical characteristics and treatment progress of IM in patients with HMs.

### Epidemiology and Risk Factors

The incidence rate of IM in patients with HMs is increasing year by year. An autopsy-based study showed that the prevalence of IM in patients with HMs increased from 0.006 cases per 100 autopsies in 1989–1993 to 0.018 cases per 100 autopsies in 2004–2008 (*p* = 0.04) [4]. Another study collected data from autopsy reports published during 2008–2013, and found that the incidence rate of invasive fungal infections in patients with HMs was 25% (711/2804), of which IM accounted for 6%, as the third commonest invasive fungal infection after invasive aspergillosis (IA) (55.5%) and invasive candidiasis (IC) (28.5%) [5]. A Spanish study reported that the prevalence of IM increased from 1.2 per 100,000 inpatients between 1988 and 2006 to 3.3 per 100,000 inpatients between 2007 and 2015, of which 52.6% had HMs [6]. From 2001 to 2006, the Transplant-Associated Infection Surveillance Network (TRANSNET) in America reported 44 IM (0.3%) of 15,820 HSCT patients [7]. A multicenter, retrospective study conducted in America found that 1133 of 962,428 HMs patients suffered from IM (0.12%) between 2007 and 2019 [8]. From 2007 to 2017, a survey of the Children’s Cancer Hospital in Egypt found 45 cases of proven IM among 13,735 hospitalized children (0.33%) who suffered from tumors [9].

There are apparent differences in the risk factors of IM among countries with geographical and economic differences. A prospective study in India between 2016 and 2017 on 465 patients diagnosed with proven IM showed that diabetes mellitus was the major risk factor associated with IM (*n* = 342, 73.5%), whereas epidemiological studies on patients with IM in Japan and North America showed that patients suffered from HMs accounted for 56.39% (75/133) and 61.2% (74/121) of the total number of patients with proven or probable IM, respectively, indicating that HMs were the most common underlying diseases in these countries [10,11,12]. Typically, those suffering from HMs have poorer prognosis than patients with other underlying diseases. An epidemiological study in France between 2005 and 2007 included 101 patients diagnosed with proven or probable IM, among whom 50 (50%) were complicated with HMs, 23 with diabetes (23%), and 18 with trauma (18%) [13]. The result showed that there was a significant difference in the mortality rate of these three types of patients infected with IM (60% vs. 32% vs. 11%, *p* = 0.008), and the mortality was higher for patients with HMs compared with patients with diabetes mellitus or with trauma.

Acute leukemia, neutropenia, steroid therapy, allo-HSCT, and graft-versus-host disease (GvHD) are factors contributing to high susceptibility to IM in patients with HMs. Table 1 collects data of risk factors for IM in patients with HMs or those undergoing HSCT from 9 studies. In a retrospective study of 32,815 patients with HMs and 1765 patients undergoing HSCT, the incidence rate of IM was highest in allo-HSCT recipients (1.19%), followed by acute lymphoblastic leukemia (0.75%) patients and acute myeloid leukemia (0.45%) patients and IM was associated with the shortest median survival time compared with IA and IC (3 months vs. 7 months vs. 7 months) [14]. Riches et al. found 6.01 cases of IM per 1000 patients who received allo-HSCT, and the high-risk factors for IM included history of *Aspergillus* infection (relative risk (RR) 4.91, *p* = 0.0007), preceding acute GVHD (RR 1.78, *p* = 0.027), and age > 50 years (RR 2.28, *p* = 0.0006) [15].

Breakthrough mucormycosis (BT-MCR) usually occurs in patients who are treated with antifungals without anti-*Mucorales* activity, mainly voriconazole and echinocandins. However, many studies found that HMs patients with high-risk factors such as active HMs, recurrent/refractory leukemia, prolonged neutropenia, and so on, will still have BT-MCR even under the prophylaxis or treatment of *Mucorales*-active antifungals, and such patients often have low survival rate and poor prognosis. By retrospectively analyzing the clinical data of 24 cases of breakthrough invasive mold infections that occurred during posaconazole (*n* = 8) or voriconazole (*n* = 16) prophylaxis among patients with HMs or undergoing transplantation (HSCT or lung transplantation) during 2009–2013 at Duke University, Lamoth et al. found that BT-MCR was one of the most common breakthrough invasive mold infections (9/24, 37.5%), of which seven cases received voriconazole and two cases received posaconazole for prophylaxis [20]. Another retrospective study of 145 HMs patients and HSCT recipients who received isavuconazole prophylaxis between 2016 and 2018 found that 12 patients (8.3%) had breakthrough invasive fungal infections including five cases of *Aspergillus fumigatus*, two cases of other *Aspergillus* species, two cases of *Mucorales*, two cases of *Fusarium* species, and one case of *Candida glabrata*, and all 12 patients had a median duration of neutropenia of 25.5 days and relapsed/refractory acute leukemia [21]. Between 2000 and 2020, 103 patients experienced BT-MCR in a single center, among whom 16 patients developed BT-MCR while on *Mucorales*-active antifungals (nine cases of isavuconazole, six cases of posaconazole, one case of AmB) and the other 87 patients developed BT-MCR while on antifungals without anti-*Mucorales* activity such as voriconazole, echinocandins, and itraconazole [22]. The 42-day mortality of patients developing BT-MCR while on *Mucorales*-active antifungals was higher than that of the remaining patients (63% vs. 25%, *p* = 0.006), and exposure to *Mucorales*-active antifungals was an independent predictor of death in patients with BT-MCR (hazard ratio (HR) 4.64, *p* < 0.001). Table 2 summarized characteristics of patients with BT-MCR.

The recent surge in cases of CAM accompanied with the pandemic of the coronavirus disease 2019 (COVID-19) made it a global health threat. As of 7 June 2021, India recorded 28,252 cases of IM, among whom 24,370 cases with a history of COVID-19 [26]. Moreover, CAM cases were also reported worldwide including in Turkey, Egypt, China, America, Iran, Spain, and so on [27,28]. Most of these patients were complicated with poorly controlled blood glucose and were treated with heavy steroids and broad-spectrum antibiotics for SARS-CoV-2 infection, which resulted in weakened immune system and highly susceptibility to *Mucorales* [28,29]. Arora et al. conducted a case–control study comparing cases diagnosed with CAM with controls who recovered from COVID-19 without developing CAM [30]. A total of 152 patients of CAM (cases), including 120 proven and 32 probable, and 200 controls were included in the study. The result showed that diabetes (92.1% vs. 28%, *p* < 0.001), poor glycemic control (90.6% vs. 51.5%, *p* < 0.001), severe COVID-19 (21% vs. 9.9%, *p* < 0.001), systemic use of steroids (65.8% vs. 48%, *p* = 0.001) were more frequently observed in cases than controls.

**Figure 1:**
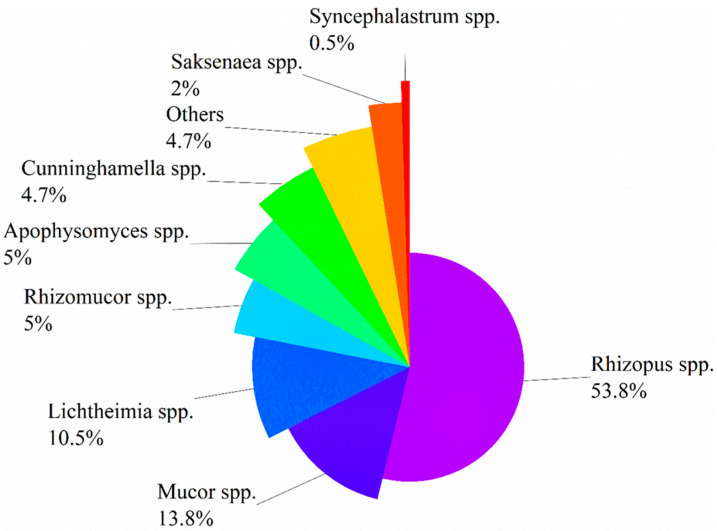
Distribution of *Mucorales* organisms causing infection.

A study in Australia on 74 patients diagnosed with proven or probable IM, among whom 36 (48.6%) were complicated with HMs, and found that *Rhizopus* spp. (20/36, 55.6%) was the most common genus of *Mucorales* organisms, followed by *Mucor* spp. (6/36, 16.7%) and *Rhizomucor* spp. (4/36, 11.1%) [31]. Another meta-analysis reported 851 patients with proven or probable IM, of which 275 (33%) were complicated with HMs, and it documented that *Rhizopus* spp. was the major pathogen of IM, followed by *Mucor* spp. (14%) and *Lichtheimia* spp. (13%) [32]. Other pathogenic genera included *Apophysomyces* spp., *Cunninghamella* spp., *Rhizomucor* spp., *Saksenaea* spp., and *Synchephalastrum* spp., but they were relatively rare. Of note, the mortality associated with *Cunninghamella* infections was remarkably higher than that caused by other genera of *Mucorales* organisms (71% vs. 44%, *p* < 0.001). The author of this review summarized the clinical data of 1568 patients with IM (whose underlying diseases included HMs, diabetes mellitus, trauma, etc.) from nine studies (which included more than 50 cases) [7,10,11,13,31,32,33,34,35], and found that the three major pathogenic genera were *Rhizopus* spp. (*n* = 778, 53.8%), *Mucor* spp. (*n* = 199, 13.8%), and *Lichtheimia* spp. (*n* = 152, 10.5%). Figure 1 illustrates the general distribution of common pathogenic genera of *Mucorales* organisms.

To sum up, HMs are the most common risk factors associated with IM in developed countries, contrasting to diabetes mellitus in developing countries. Factors such as acute leukemia, neutropenia, allo-HSCT, and steroid therapy make patients with HMs highly susceptible to IM. BT-MCR still occurs in patients with active HMs, recurrent/refractory leukemia, and prolonged neutropenia under the prophylaxis of *Mucorales*-active antifungals, and such patients often have relatively high mortality and poor prognosis. *Rhizopus* spp. is the most common pathogen of IM, followed by *Mucor* spp. and *Lichtheimia* spp.

## 2. Clinical Manifestations

**Figure 2:**
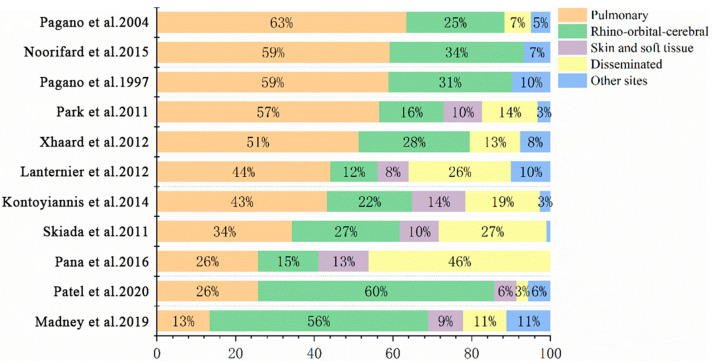
Sites of IM in patients with HMs [2,3,7,9,10,11,13,17,19,33,36].

**Figure 3:**
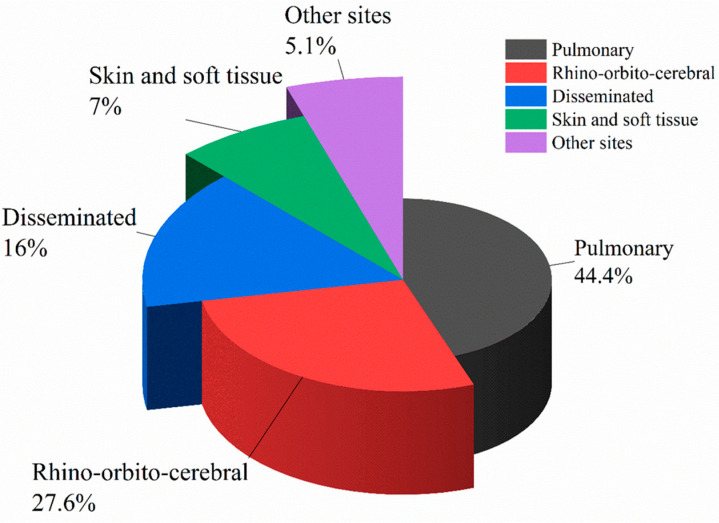
Proportion of various types of IM differentiated by sites in patients with HMs.

According to anatomic localizations and clinical manifestations, IM is divided into the following six clinical types: pulmonary mucormycosis (PM), rhino-orbital-cerebral mucormycosis (ROCM), disseminated mucormycosis, cutaneous/soft tissue mucormycosis, gastrointestinal mucormycosis and other rare forms, such as renal infection, endocarditis, osteomyelitis, peritonitis, and so on. CAM and IM in patients with diabetes mellitus usually manifest as ROCM. In contrast, most scholars believe that PM and disseminated mucormycosis occurs most often in patients with HMs. By analyzing cases of proven or probable IM in pediatric (≤19 years) patients, Pana et al. found that among 29 patients with PM, 22 had HMs, which were independently correlated with PM (odds ratio (OR) 4.4, *p* = 0.01) [36]. However, Slavin et al. analyzed 162 patients with NAIMIs (145 proven and 17 probable), of whom 74 (45.7%) were IM, and found HMs independently predicted disseminated infections (OR 2.7, *p* = 0.03) [35]. A study in Europe between 2005 and 2007 on 102 patients with HMs who were complicated with proven or probable IM indicated that the major sites of IM in these patients were pulmonary (*n* = 35, 34%) and disseminated (*n* = 28, 27%) [33]. On the other hand, in a retrospective study that included 20 patients with HMs who were diagnosed with proven IM, the most frequently involved site of IM was paranasal sinuses (*n* = 19, 95%) and PM only occurred in one patient (5%) [37]. We collected and analyzed infection sites of 604 cases of IM in HMs patients and HSCT patients from 11 studies [2,3,7,9,10,11,13,17,19,33,36] which included more than 25 cases and had detailed records of involved sites of IM (some patients have two or more infection sites). The result showed that the most common form of IM in patients with HMs or patients undergoing HSCT is PM (44.4%), followed by ROCM (27.6%) and disseminated mucormycosis (16%), which is shown in Figure 2 and Figure 3.

Fever is the most common clinical manifestation of IM, and almost all patients with IM have varying degrees of fever. Table 3 summarized clinical and imaging manifestations of PM, rhino-orbital mucormycosis, and central nervous system (CNS) mucormycosis. The triad of “cough, dyspnea, and chest pain” is a relatively specific sign of PM. Imaging manifestations of PM include exudation, cavity, ground-glass lesion, consolidation, pleural effusion, atelectasis, halo sign, reverse halo sign, air-crescent sign, etc. A retrospective study on HMs patients with IM or IA, including 59 proven IM patients and 541 proven IA patients showed that, compared with patients with IA, patients with IM had a significantly higher frequency of local pain syndrome (53% vs. 5%, *p* = 0.0001), hemoptysis (32% vs. 6%, *p* = 0.001), pleural effusion (53% vs. 7%, *p* = 0.003), destructive lesions (38% vs. 8%, *p* = 0.0001), and “reverse halo” sign (17% vs. 3%) [38]. In addition, although neutropenia and lymphocytopenia represented the major risk factors in both groups, patients with IM had a longer duration of severe neutropenia (30 vs. 14 days, *p* = 0.0001) and lymphocytopenia (25 vs. 14 days, *p* = 0.001) [38]. Both Jung et al. and Chamilos et al. compared the CT findings of PM and IA in patients with HMs, and they found the frequency of “reverse halo” sign (54% vs. 6% *p* < 0.001), multiple (≥10) nodules (64% vs. 18%, *p* = 0.02) and pleural effusion (63% vs. 33%, *p* = 0.1) in patients with PM were significantly higher than those in patients with IA [39,40]. The CT findings of patients with rhino-orbital mucormycosis include thickened oedematous mucosa, opacification or obliteration of paranasal sinuses and bony destruction, while MRI shows non-enhancing mucosal tissue within the involved sinuses and turbinates, also known as “black turbinate sign” [41,42]. CNS mucormycosis can be isolated, but it can also occur due to contiguous spread from the paranasal sinuses and orbits or hematogenous spread [43]. Compared with CT, MRI can identify intracranial infections, such as intraventricular “fungus balls”, thrombosis of intracranial arteries, the inflammatory alterations of the cavernous sinus and the involvement of adjacent structures (such as meninges), more sensitively and accurately [44].

In conclusion, the most common form of IM in patients with HMs is PM, followed by ROCM and disseminated mucormycosis. “Reverse halo” sign, multiple (≥10) nodules, and pleural effusion are relatively specific imaging manifestations of patients with PM, while the imaging manifestations of ROCM patients are not very specific. MRI can better reflect the intracranial infections than CT.

## 3. Prognosis

The prognosis of IM in patients with HMs is usually poor. In a study of 70 patients with HMs who had proven (45 cases) or probable (25 cases) IM, Chamilos et al. found that the 4-week and 12-week mortality of 35 patients who received AmB delayed treatment (≥6 days after symptom onset) was twice that of 35 patients receiving early treatment (<6 days after symptom onset) (4-week mortality: 35.1% vs. 66.6%, *p* = 0.006; 12-week mortality: 48.6% vs. 82.9%, *p* = 0.03) [45]. The authors also found that delayed AmB-based treatment (OR 8.1, *p* = 0.008), the presence of an active HM (OR 12.2, *p* = 0.003), and monocytopenia (OR 5.5, *p* = 0.01) were independent risk factors of death, while salvage posaconazole-based treatment (OR 0.1, *p* = 0.01) and neutrophil recovery (OR 0.07, *p* = 0.009) were independently associated with survival. A retrospective study on 21 cases of PM (11 proven, 10 probable) in allo-HSCT recipients showed that the survival time of patients with hemoptysis was significantly shorter than that of patients without hemoptysis (*p* < 0.05), which was the same with patients with GVHD and without GVHD (*p* = 0.043) [46]. A meta-analysis of 1544 cases of proven or probable PM showed that although the mortality of patients with PM decreased significantly over time (72.1% vs. 58.3% vs. 49.8% for studies before 2000, 2000–2009, and 2010–2020, respectively, *p* = 0.00001), the mortality during 2010–2020 was still close to 50% [47]. The authors also found disseminated mucormycosis had a higher risk of death than isolated PM, and combined medical–surgical therapy reduced mortality compared to medical treatment alone [30.9% (43/139) vs. 69.3% (228/329)]. To sum up, neutrophil recovery, localized mucormycosis, and early combined medical-–surgical therapy are associated with favorable prognosis, while hemoptysis, GvHD, disseminated mucormycosis, uncontrolled underlying diseases, and delayed AmB treatment are associated with poor prognosis.

## 4. Diagnosis

Early diagnosis and timely effective treatment are keys to improving the survival probability of IM in patients with HMs. The accurate diagnosis of IM relies on a series of a high index of suspicion, assessment of presenting signs and symptoms, radiographical studies, cultures and direct examinations of clinical specimens, and histopathology. Notably, there are no commercially available biomarkers to identify this disease. β-D-glucan and galactomannan assays do not detect antigen components of *Mucorales* cell wall.

Generally, histopathology and culture are gold standards for IM diagnosis. The diagnosis of proven IM can be classified as: a. histopathologic, cytopathologic, or direct microscopic examination of a specimen obtained by needle aspiration or biopsy; b. positive culture from a sterile site or blood; c. detection of *Mucorales* DNA by PCR combined with DNA sequencing when *Mucorales* hyphae are seen in formalin-fixed paraffin-embedded tissue. Probable IM diagnosis is based on corresponding host factors (such as neutropenia, allo-HSCT, HMs, etc.), imaging features (including “reverse halo” sign, multiple (≥10) nodules on pulmonary CT scan, vessel occlusion on CT pulmonary angiography or sinusitis, bony destruction on cranial CT or MRI) and culture or microscopical detection from sputum, bronchoalveolar lavage (BAL), bronchial brush, or aspirate [48].

Recently, molecular-based diagnostic assays were developed rapidly and were recommended as valuable add-on tools that complement conventional diagnostic procedures. Furthermore, molecular analysis can identify *Mucorales* isolates at the species level accurately, compensating for the shortage of phenotypic differentiation. Many studies proved the diagnostic value of molecular detection of *Mucorales* DNA in tissue specimens [49,50,51]. Additionally, there are also several molecular methods were evaluated for direct detection in body fluid specimens. Lengerova et al. validated a PCR followed by high-resolution melt analysis (PCR/HRMA) to detect *Rhizopus* spp., *Rhizomucor pusillus*, *Lichtheimia corymbifera*, and *Mucor* spp. in BAL samples from immunocompromised patients who were at risk of invasive fungal disease [52]. The sensitivity and specificity of PCR/HRMA were 100% and 93%, respectively, and the negative predictive value reached 99%. Springer et al. tested the sensitivity of the PCR using the cell pellet or the supernatant fraction of the BAL fluid [53]. They collected 99 BAL specimens from 96 hematology patients with or without allo-HSCT. As a result, they found testing of the combination of the cell pellet and the supernatant fraction generated higher sensitivity than testing of single fraction of BAL. Both studies suggested a role for PCR applications in BAL samples for the diagnosis of PM in hematological patients.

However, since patients with HMs or HSCT sometimes suffer from thrombocytopenia or coagulopathy, invasive procedures such as biopsy and BAL are frequently not feasible. Therefore, studies attempted to direct at molecular-based diagnosis from blood and urine [54,55,56,57]. Because of the angio-invasive nature of IM, the load of circulating *Mucorales* DNA in serum was found to be significantly higher than those of IA, indicating that detection of serum *Mucorales* DNA can help to anticipate the diagnosis of IM and trigger early targeted antifungal treatment [58]. Millon et al. evaluated the performance of *Mucorales* quantitative PCR (qPCR) targeting *Lichtheimia*, *Rhizomucor*, and *Mucor/Rhizopus* on serum samples of 232 patients with suspicion of invasive mold disease [54]. Positive *Mucorales* qPCR serum samples were obtained from 23 patients among 27 patients with proven/probable IM confirmed by histopathological examination and/or positive culture. The sensitivity was 85.2%, specificity 89.8%, and positive and negative likelihood ratios 8.3 and 0.17, respectively. The first *Mucorales* qPCR-positive serum was observed a median of 4 days before sampling of the first mycological or histological positive specimen and a median of 1 day before the first imaging was performed. Mercier et al. also assessed the diagnostic property of a *Mucorales* PCR assay for the detection of *Mucorales* DNA on serial blood samples from patients with culture-positive IM [56]. They found that a positive PCR assay result preceded a positive culture result by up to 81 days with an overall sensitivity of 0.75.

Xu et al. retrospectively analyzed the results of plasma cell-free DNA next-generation sequencing (NGS) performed on 347 specimens collected from hematological patients who were suspected of infections and evaluated its diagnostic performance [59]. The overall positive detection rate of plasma cell-free DNA sequencing was significantly higher than that of conventional microbiological tests (72.6% vs. 31.4%, *p* < 0.001). Of note, *Mucorales* was only detected by the NGS method. Hill et al. also retrospectively assessed the diagnostic performance of plasma cell-free DNA NGS in 114 HSCT recipients with pneumonia after HSCT [60]. Among 75 patients with proven/probable pulmonary mold infections, plasma cell-free DNA NGS generated a 51% sensitivity with high specificity (95% CI, 82–100%) and up to 100% positive predictive value. The above evidences show that the detection of serum *Mucorales* DNA is an appealing non-invasive diagnostic tool with high sensitivity and specificity.

## 5. Therapy

For patients diagnosed with proven, probable, or possible IM, risk factors should be eliminated as early as possible, including treating underlying diseases, reducing or stopping corticosteroids and other immunosuppressive treatments, stopping deferoxamine, and controlling blood sugar of patients with diabetes mellitus in the normal range. The European Confederation of Medical Mycology (ECMM) strongly recommended early complete surgical treatment for patients with surgical tolerance, first-line treatment with Liposome amphotericin B (L-AmB) for patients without preexisting renal compromise, and intravenous formulations of isavuconazole or posaconazole for patients with impaired renal function [61]. Intravenous formulations or tablets of isavuconazole and posaconazole can also be used as salvage treatment for patients who are refractory or intolerant to first-line treatment of L-AmB.

### 5.1. First-Line Treatment

#### 5.1.1. Surgical Treatment

ECMM guidelines and ECIL-6 guidelines strongly recommended radical surgical debridement with margins clear of infection for the treatment of IM if conditions permit [61,62].

Claustre et al. retrospectively analyzed the clinical data of 74 patients with IM (48 proven, 26 probable) in 16 ICUs in France from 2008 to 2017, among whom 41 were complicated with HMs and 27 (36.5%) received surgical intervention [63]. Finally, 21 patients survived to ICU stay, with an overall survival in ICU of 28.4%. The authors compared the survivors of ICU stay with non-survivors and found that strategies including a surgical therapeutic management was associated with a better survival (*p* = 0.03). Since patients with HMs had poorer prognoses, with only 7 patients (17.1%) surviving to ICU stay, the authors focused on this group of patients and analyzed further. Unsurprisingly, they found curative surgery was closely related to the survival of IM in patients with HMs (OR = 0.71, *p* < 0.001). A meta-analysis included 851 adult patients with proven (750 cases) or probable (101 cases) IM and 275 (32%) of them were complicated with HMs [64]. According to their treatment data, antifungal therapy in combination with surgery was the most commonly prescribed treatment (476/815, 58%) and this combination protocol was associated with significantly lower 90-day mortality compared to treatment with antifungals alone [144/476 (30%) versus 131/226 (58%), *p* < 0.001].

A multicenter study on 39 cases of IM (33 proven, 4 probable, 2 possible) in children with HMs in Israel reported that the 12-week mortality of the 26 children who received two or more debridement operations or extended resections was significantly lower than that of the remaining 13 children without debridement (16% vs. 71%, *p* < 0.001) [65]. By retrospectively analyzing the clinical data of 74 patients with proven ROCM, Cag et al. found that among the 56 patients who received surgical treatment, the proportion of survived patients was significantly higher than that of dead patients [67.9% (38/56) vs. 32.1% (18/56), *p* = 0.001], and multivariate logistic analysis demonstrated that no surgical debridement was independently associated with an increased risk of death (OR = 5.92, *p* = 0.050) [66]. Furthermore, surgical treatment can help control infection and prevent infection from spreading, thus creating conditions for follow-up HSCT in patients with HMs [67,68]. It can be seen that surgical treatment plays an important role in improving the survival rate and prognosis of patients with IM.

There are also studies suggesting that radical surgical debridement may not be able to control IM and improve the survival of patients with HMs if their underlying diseases are not relieved. In a retrospective study on 22 patients with proven (17 cases) or probable (five cases) ROCM who received surgical treatment, 14 (82.4%) of 17 patients who were complicated with diabetes mellitus or other diseases achieved local control of ROCM, whereas all five patients complicated with HMs died because the underlying diseases were not controlled [69]. The univariate analysis in the study showed that HMs were significantly associated with mortality of patients with ROCM (*p* < 0.0001). Therefore, the premise of successful surgical treatment of IM is that the underlying diseases were well controlled.

Patients with HMs often cannot tolerate surgery due to serious underlying diseases, coagulation dysfunction and disseminated mucormycosis; so, the role of surgery is limited. However, surgical treatment is still recommended for patients who have localized IM and those who can tolerate surgery. On the one hand, surgical treatment can timely remove necrotic tissue, prevent the spread of the infection, reduce the risk of infection recurrence in the process of follow-up chemotherapy, and provide a basis for etiological diagnosis; on the other hand, it creates conditions for follow-up HSCT of patients with HMs.

#### 5.1.2. Liposomal Amphotericin B (L-AmB)

L-AmB has a strong anti-*Mucorales* activity whose minimum inhibitory concentration against most strains of *Mucorales* in vitro is less than 1 µg/mL. Its nephrotoxicity is lower than that of traditional AmB (AmB deoxycholate). Due to its strong anti-*Mucorales* activity and good tolerance, L-AmB was recognized as the first choice for treatment of IM. L-AmB combined with surgery is strongly recommended as the first-line treatment of IM by ECMM guidelines and ECIL-6 guidelines [61,62].

A retrospective study included 92 patients with proven PM among whom 82 received AmB therapy, and found patients treated with AmB deoxycholate (*n* = 41) had a poorer prognosis than those treated with lipid AmB (*n* = 41) (mortality rate: 36.6% vs. 9.8%), which indicated that lipid AmB had less toxicities and better tolerance [70]. Pagano et al. conducted a retrospective study on 59 cases of IM in patients with HMs and found that treatment with L-AmB was significantly correlated with recovery from IM (risk ratio = 0.50, *p* < 0.001) [19]. For patients with localized IM in sinus, brain or lung, local administration of L-AmB can be a good choice of treatment which significantly reduced toxicities secondary to antifungal therapy. There were many case reports that showed that nasal irrigation and aerosol inhalation of L-AmB played an important role in achieving local control of IM while avoiding toxicities secondary to antifungal therapy to the maximum extent [71,72,73]. To sum up, the early application of L-AmB is essential to reducing the mortality of IM in patients with HMs and improving their quality of life.

ECMM guidelines and ECIL-6 guidelines recommended that the commonly administration dose of L-AmB for treatment of IM is 5–10 mg/kg, while if CNS involved, 10 mg/kg/d is better [61,62]. Of note, the full daily dose should be given from the first treatment day instead of increasing over several days. In a prospective pilot study of high-dose (10 mg/kg/day) L-AmB for the initial treatment of IM, 34 patients with IM (29 proven, 5 probable) were included, among whom 18 (53%) were complicated with HMs [74]. The result showed the response rates at 4 and 12 weeks were 36% (*n* = 12) and 45% (*n* = 14) and the mortality rates at 12 and 24 weeks were 38% (*n* = 13) and 53% (*n* = 18), respectively. The main adverse reaction associated with the drug was the doubled serum creatinine in 16 (40%) patients, but returned to normal levels within 12 weeks in 10/16 (63%). Therefore, L-AmB is recommended to be administered in sufficient dose, but if high-dose L-AmB is needed, the risk of nephrotoxicity in patients should be weighed before the drug is administrated. In short, the key is to individualize the medication.

#### 5.1.3. Isavuconazole

Isavuconazole, the active moiety of the water-soluble prodrug isavuconazonium sulfate, is a new generation of broad-spectrum triazoles with good pharmacokinetic characteristics. Isavuconazole was approved as one of the first-line antifungals of IM in the United States, while it was approved to treat patients with IM who are not suitable for AmB in Europe. ECMM guideline and Infectious Diseases Working Group of the German Society of Hematological Oncology (AGIHO DGHO) also recommended it for first-line treatment of IM [61,75].

In VITAL study, 37 patients with proven (32 cases) or probable (5 cases) IM who were treated with isavuconazole were included, among whom 22 (59%) patients were complicated with HMs. At the end of the treatment, the complete and partial response rate was 31% (*n* = 11), and the main adverse events during the treatment were gastrointestinal reactions (nausea, vomiting, etc.). Marty et al. matched the 21 patients who were treated with isavuconazole as initial treatment with 33 patients who initially received AmB-based therapy, and found day-42 crude all-cause mortality was similar in these two groups of patients (33% vs. 39%, *p* = 0.775) and there was also no significant difference in day-84 survival rate between the two groups (57% vs. 50%, *p* = 0.653) [76]. The study showed that isavuconazole had similar efficacy to AmB with regard to treatment of IM, but isavuconazole has no dose-dependent nephrotoxicity, and so, it can be used as one of the first-line antifungals for patients with IM or as an alternative antifungal for those who are intolerant of AmB. In a multicenter study, which included 108 patients with proven or probable IM, of whom 50 (46.3%) were complicated with HMs, the day-42 all-cause mortality of patients receiving isavuconazole for initial treatment, isavuconazole for salvage treatment and other antifungal drugs were 33.3% (14/42), 20.0% (4/20), and 41.3% (19/46), respectively, and the day-84 all-cause mortality of these patients were 40.5% (17/42), 25% (5/20), and 50% (23/46) [77]. The result showed the mortality of patients treated with isavuconazole was lower than that of patients treated with other antifungals, indicating that isavuconazole was safe and effective for treatment of IM in patients with HMs.

In addition, a recent retrospective study showed that isavuconazole can be used as an effective antifungal for the treatment of CNS mucormycosis. The study was conducted on the clinical data of 36 patients with proven (33 cases) or probable (three cases) CNS invasive mold infections, among whom 11 (30.6%) suffered from CNS mucormycosis [78]. After a median duration of 103.5 days of isavuconazole treatment, 21 patients (58.3%) achieved complete or partial clinical response at the end of treatment, and the overall survival rates at day 42 and day 84 were 80.6% (*n* = 29) and 69.4% (*n* = 25), respectively. However, since it was a retrospective analysis and with a small research population, the result must be confirmed with larger studies.

Compared with other azole drugs, isavuconazole not only has a broad antifungal spectrum, but also has higher safety. A single center retrospective study including 100 patients to compare clinically relevant safety and efficacy outcomes in real world patients treated with isavuconazole, voriconazole, or posaconazole showed that the incidences of both composite safety outcome (*p* = 0.028) and QTc prolongation (*p* = 0.037) in patients treated with isavuconazole were significantly lower than that in patients treated with voriconazole or posaconazole [79]. According to another single center retrospective analysis, 23 patients with HMs switched to isavuconazole for prophylaxis or treatment of invasive fungal infections because of toxicities caused by posaconazole (20 patients had azole-induced hepatotoxicity, and three patients had grade 3/4 grade QTc prolongation on ECG) [80]. After switching to isavuconazole, grade 3/4 elevations in liver function tests (total bilirubin, alkaline phosphatase, alanine aminotransferase (ALT), aspartate aminotransferase (AST)) of the 20 patients gradually decreased to normal range, the ECG of the three patients with QTc prolongation returned to normal limits as well, and the day-30 and day-60 mortality rates of all these patients were 22% (*n* = 5) and 30% (*n* = 7), respectively.

The common clinical administration scheme of isavuconazole is loading dose 372 mg of isavuconazonium sulfate (equivalent to 200 mg of isavuconazole) three times daily for 2 days, orally or intravenously, and then changed to a maintenance dose 372 mg of isavuconazonium sulfate once daily. A phase 3 study demonstrated that the pharmacokinetics of isavuconazole had a linear relationship with little individual difference, and its efficacy and safety were not observed to be dependent on the drug concentration [81]. Therefore, therapeutic drug monitoring is not routinely required for isavuconazole. However, real-life studies demonstrated inter-patient variability in isavuconazole exposure (coefficients of variation of 51% for area under the plasma concentration-time curve and 59% for trough plasma concentration) [82] and relationships between isavuconazole blood concentrations and side effects [83]. Bolcato et al. and Höhl et al. also found that factors such as body mass index ≥ 25, higher sepsis-related organ failure assessment score, aspartate aminotransferase, and protein levels were associated with isavuconazole exposure variability [84]. Further studies are needed to investigate the variability of isavuconazole trough concentrations and its role on drug efficacy and/or toxicity.

#### 5.1.4. Posaconazole Intravenous Formulation/Delayed Release Tablet

Salmanton Garcia et al. performed a case-matched analysis in patients with proven/probable IM in order to compare the efficacy and safety of posaconazole new formulations (intravenous formulation or delayed release tablet) with AmB or posaconazole oral suspension for treatment of patients with IM [85]. The results showed that in first-line treatment group, compared with patients receiving AmB monotherapy, patients who received posaconazole new formulations monotherapy (*n* = 5) or combined with AmB (*n* = 18) had higher favorable response rate [80.0% (4/5) vs. 40.0% (6/15), 50.0% (9/18) vs. 38.0% (19/50)], and lower mortality [40.0% (2/5) vs. 60.0% (9/15), *p* = 0.617; 50.0% (9/18) vs. 60.0% (30/50), *p* = 0.580] at the end of treatment. When new formulations of posaconazole were used as salvage treatment, a favorable response was reported in 77.2% (17/22) of the patients and in 66.7% (30/45) of the matched cohort who received posaconazole oral suspension, and the mortality at the end of treatment of patients who were treated with posaconazole new formulations was lower as compared to controls [18.2% (4/22) vs. 33.3% (15/45), *p* = 0.255]. Although the difference was not statistically significant, treating with posaconazole new formulations showed a higher favorable response rate and survival rate. It can be seen that posaconazole new formulations had good efficacy and safety as the first-line treatment or salvage treatment of IM in patients with HMs, but due to the limited sample size of the study, the exact efficacy of the drug needs to be further verified.

The recommended dose of posaconazole intravenous formulation and delayed release tablet was 300 mg twice a day on the first day, followed by 300 mg once a day, regardless of diet. Although posaconazole intravenous formulation and tablet have higher bioavailability than that of oral suspensions and are easier to reach the target concentration, therapeutic drug monitoring is still recommended to optimize administration regimens [86,87]. Patel et al. evaluated posaconazole serum levels of 29 patients who received posaconazole delayed release tablet as first-line or alternate therapy to treat IM during the period of COVID-19 pandemic, and identified seven patients (24.1%) with sub-therapeutic posaconazole trough level [88]. Therefore, the author suggested that the trough level of posaconazole should be monitored on the fourth day of posaconazole delayed release tablet treatment.

#### 5.1.5. Antifungals Combination Therapy

IM of patients with HMs is usually difficult to control and dose-dependent nephrotoxicity often makes patients unable to tolerate high doses of AmB; thus, these patients always have poor prognosis. However, there are many studies that proved the efficacy of combined antifungals in the treatment of IM. Miller et al. conducted a retrospective study on 64 cases of IM (47 proven, 17 probable) in patients with HMs, among whom 28 (44%) were initially treated with AmB monotherapy, 16 (25%) were initially treated with AmB+posaconazole new formulations, and 5 (8%) were initially treated with AmB+isavuconazole [89]. The result showed that compared with AmB monotherapy, initial treatment with AmB plus posaconazole new formulations or isavuconazole was associated with a trend toward lower treatment failure (43% vs. 64%, *p* = 0.136), although the difference was not statistically significant. Pagano et al. reported the efficacy of the combination of L-AmB and posaconazole oral suspension in the treatment of 32 cases of IM (20 proven, 12 probable) in patients with HMs [90]. The combination treatment was administered as first-line treatment to three patients, as second- or third-line treatment in 29 patients who lacked a response to antifungal monotherapy (mostly AmB). After a median of 32 days of combination treatment, 18 patients (56%) achieved complete (*n* = 11) or partial response (*n* = 7) and 5 patients had stable disease, and none of the patients had to stop antifungal treatment because of drug-related toxicity. The result indicated that a combined antifungal treatment with L-AmB+posaconazole might be a useful treatment protocol for IM in patients with HMs.

Skiada et al. conducted an epidemiological study on 230 patients with proven (112 cases) or probable (118 cases) IM between 2005 and 2007 in Europe, and they found AmB combined with posaconazole oral suspension (OR 0.09, *p* = 0.003) or AmB combined with posaconazole oral suspension and other antifungals (OR 0.05, *p* = 0.029) could significantly decrease the risk of death in patients with IM [33]. By retrospectively analyzing the clinical data of 23 patients with NAIMIs, of whom 10 suffered from IM, Jenks et al. also reported that the mortality of patients receiving treatment of L-AmB+posaconazole was significantly lower than that of patients receiving L-AmB monotherapy [3/13 (23%) vs. 9/10 (90%), *p* = 0.003] [91].

To sum up, the combination of antifungals showed good therapeutic effect. Patients who have refractory IM, or who do not respond to monotherapy or who cannot tolerate the toxicities associated with high-dose L-AmB monotherapy, can choose antifungals combination therapy.

### 5.2. Salvage Therapy

Patients who are with recurrent/refractory IM or intolerant to initial treatment of L-AmB need to turn to second-line treatment schemes. The ECMM guideline strongly recommended that isavuconazole, posaconazole intravenous formulations or tablets could be used as salvage treatment for patients with IM [61].

In a case report, four pediatric patients with proven IM, of whom three were complicated with HMs, received isavuconazole as salvage treatment [92]. Isavuconazole was administered alone or combined with other antifungal agents in three of them for refractory disease, and in one after intolerance to another antifungal drug. All four patients achieved complete clinical, radiologic, and mycologic responses after a median of 2.5 months of isavuconazole therapy combined with surgery, and no adverse events related to isavuconazole were observed [92]. In addition, the author retrospectively analyzed the clinical data of eight HMs children who were complicated with IM, of whom six received salvage treatment with isavuconazole and six received initial treatment with the combination of isavuconazole and AmB, and all these children survived. This study suggested that isavuconazole could be used as a salvage treatment for IM in children with HMs with good efficacy and safety.

Van Burik et al. conducted a retrospective study to evaluate the activity of posaconazole oral suspension for salvage treatment of IM, including 91 patients with proven (69 cases) or probable (22 cases) IM, among whom 48 were complicated with HMs [93]. These patients were either refractory to prior antifungal treatment (*n* = 81) or intolerant of such treatment (*n* = 10) and received posaconazole oral suspension for salvage treatment. The result showed that the complete and partial response rate of these patients at 12 weeks after treatment initiation was 60% (*n* = 55), and the other 21% (*n* = 19) of patients had stable disease. Fortun et al. also conducted a multicenter observational study on 67 patients with IFI (14 proven, 42 probable, 11 possible) to investigate the safety and efficacy of posaconazole in its different forms of administration (intravenous formulation, tablet and oral suspension) in the salvage treatment of IFI [94]. Among these patients, nine suffered from IM, and the clinical response at 3 and 12 months of posaconazole therapy were 55.5% (*n* = 5) and 55.5% (*n* = 5), respectively.

In conclusion, for patients who can tolerate surgical treatment, early debridement and removal of involved lesions can significantly improve the survival rate. However, surgery is not applicable to all patients. Patients with critical underlying diseases, coagulation disorders, and disseminated mucormycosis often cannot tolerate radical surgery. L-AmB is still the drug of choice for treatment of IM, but the intravenous formulations and tablets of both isavuconazole and posaconazole are also effective antifungals of IM. Patients who are refractory to monotherapy or cannot tolerate toxicities associated with high doses of L-AmB can try combination therapy of antifungals. Patients who are refractory or intolerant to L-AmB initial therapy can turn to salvage treatment with isavuconazole or posaconazole.

### 5.3. Other Adjuvant Treatment

#### 5.3.1. Iron Chelators

The increase in serum iron concentration and the application of deferoxamine are important risk factors for HMs patients infected with *Mucorales*. Therefore, stopping deferoxamine and reducing serum iron concentration (using deferiprone and deferasirox) may be beneficial to the treatment of IM. Chitasombat et al. reviewed and analyzed six patients with IM who were treated with deferiprone, of which 5 were complicated with HMs [95]. The patients were treated with polyenes combined with echinocandins for first-line treatment, posaconazole for step-down treatment, deferiprone for adjuvant treatment, and there were no serious deferiprone-related toxicities. In general, deferiprone was well tolerated, and four patients (67%) achieved complete or partial response after 12 weeks of treatment.

#### 5.3.2. Granulocyte Macrophage Colony-Stimulating Factor

As illustrated above, Chamilos et al. found that neutrophil recovery was significantly related to the recovery of IM in patients with HMs [45]. Kontoyiannis et al. also found that the favorable response rate of IM treatment in HMs patients with recovered neutrophil count was significantly higher than that in patients without neutrophil recovery (5/12 vs. 0/9, *p* = 0.01) [16]. It can be seen from the above studies that reversing the status of immunosuppression and restoring immune cell count and function are essential for the treatment of IM in patients with HMs.

In a study on proven IM in children with HMs, in addition to conventional surgery and antifungal treatment, all 11 children received granulocyte-stimulating factors after the onset of IM until recovering normal white blood cell count, and eight children (72.7%) survived finally [96]. Sahin et al. reported a case of PM in a patient with HM who received combined treatment of L-AmB and granulocyte-macrophage colony-stimulating factor because of chronic neutropenia, and achieved complete response after 6 months of therapy [97]. Garcia-Diaz et al. also reported three cases of ROCM patients who were successfully treated with granulocyte macrophage colony-stimulating factor combined with traditional surgery and antifungal drugs [98].

All in all, the recovery of immune function is crucial to the treatment of IM. Granulocyte macrophage colony-stimulating factor, granulocyte-stimulating factors, or interferon-γ can be used when immune cells’ count or function of patients with IM are seriously abnormal, so as to help to restore normal immune function and optimize the effect of antifungal treatment.

#### 5.3.3. Hyperbaric Oxygen

Research shows that hyperbaric oxygen can also be used as an effective adjuvant treatment for IM. Aguiar et al. included seven patients with refractory bacterial or fungal infections in their study, three of whom suffered from ROCM [99]. Except for surgery and anti-infection therapy, all patients received hyperbaric oxygen therapy and all their infections were cured. In a retrospective study, 14 patients with IM or IA received hyperbaric oxygen therapy while receiving surgery and antifungal therapy, and at the end of the treatment, seven patients (50%) survived without any adverse event related to hyperbaric oxygen therapy (Segal et al., 2007) [100]. Yohai et al. found that four of eighteen (22%) patients with ROCM who received standard treatment (surgery+AmB) survived, while five of six patients (83%) who received standard treatment combined with hyperbaric oxygen therapy survived (*p* = 0.0285) [101]. Hyperbaric oxygen is known to inhibit the growth of fungi and promote tissue healing. The above research also proved that it can be an effective adjuvant treatment for patients with IM.

### 5.4. Novel Antifungal Drugs

Since currently available antifungals against IM are limited, researchers focused on discovering or synthesizing new promising agents. Fosmanogepix is the prodrug of manogepix, a broad-spectrum investigational antifungal agent that inhibits inositol acyltransferase, thereby preventing GPI-anchored protein maturation. Gebremariam et al. assessed the activity of fosmanogepix in neutropenic murine PM models infected with *R. arrhizus var. delemar* and *R. arrhizus var. arrhizus*, in which the minimum effective concentration (MEC) values of manogepix were low (0.25 μg/mL for *R. arrhizus var. delemar*) and high (4.0 μg/mL for *R. arrhizus var. arrhizus*) [102]. The result showed that treatment with 78 mg/kg or 104 mg/kg doses of fosmanogepix resulted in significantly improved survival rate and prolonged median survival time as compared to the placebo control. Fosmanogepix treatment also significantly reduced the fungal burdens in both lungs and the brain. Of note, the efficacy of fosmanogepix in reducing tissue fungal burden and prolonging survival of mice was found comparable to isavuconazole for the treatment of IM.

Oteseconazole is a novel metalloenzyme inhibitor which prevents the synthesis of ergosterol through selective inhibition of fungal lanosterol 14α-demethylase. Gebremariam et al. compared the efficacy of the oteseconazole with L-AmB for the treatment of IM in immunosuppressed mice caused by *R. arrhizus var. arrhizus* [103]. The result showed that oteseconazole was as effective as L-AmB in reducing lung and brain fungal burdens and improving survival rate of immunosuppressed infected mice. Jawsamycin is an oligocyclopropyl-containing natural product with broad activity against fungi. Fu et al. found that Jawsamycin exhibited in vitro antifungal activity against *Mucorales* fungi including *Rhizopus oryzae* (MEC ≤ 0.008 μg/mL), *Absidia corymbifera* (MEC ≤ 0.008 μg/mL), and *Mucor circinelloides* (MEC = 0.016 μg/mL), which were generally insensitive to current licensed antifungal agents [104]. Furthermore, the antifungal drug also demonstrated in vivo activity in a mouse model of PM due to *Rhyzopus delemar* infection, in which jawsamycin significantly improved overall survival rate versus placebo-treated mice (45% vs. 10%, *p* = 0.001), and reduced the fungal burdens in both lung and brain by one log compared to placebo mice.

Recently, a novel triazole, PC1244, was also reported to be effective against *Lichtheimia corymbifera*, *Mucor circinelloides*, *Rhizomucor pusillus*, and *Rhizopus oryzae* with minimal inhibitory concentration (MIC) 0.25–2 μg/mL which was more effective than voriconazole and posaconazole (MIC > 8 μg/mL) [105]. Elfiky reported that sofosbuvir, an antiviral drug which targets RNA-dependent RNA polymerase (RdRp) virus, could bind to *Rhizopus oryzae* RdRp and SARS-CoV-2 RdRp, thus having the potential to exert dual inhibition against SARS-CoV-2 and *Rhizopus oryzae* infections [106].

Although the development of these novel drugs is inspiring, data are limited to in vitro or animal model studies, and large-scale clinical investigations are needed.

## 6. Prophylaxis

Despite improvements in diagnosis and treatment, IM-associated mortality, especially in patients with HMs, remains high. Therefore, antifungal prophylaxis seems to be an effective strategy to prevent the development of IM.

Both ECMM and AGIHO DGHO guidelines recommended primary antifungal prophylaxis with posaconazole in neutropenic patients [61,107]. Both Cornely et al. and Ullmann et al. compared the efficacy of posaconazole oral suspension with that of fluconazole as prophylaxis for patients with HMs and/or GvHD [108,109]. The two studies had similar results: no case of IM was observed in patients who received posaconazole, while one case of IM in each study was observed in patients who received fluconazole. In addition, Duarte et al. and Cornely et al., respectively, recruited 54 and 210 patients with HMs in their studies to investigate the prophylactic efficacy of posaconazole tablet in neutropenic patients who were at high risk for IM, and neither of them found BT-MCR in their cohorts [110,111].

With broad antifungal spectrum and good tolerance, isavuconazole was also recommended as an effective prophylactic antifungal against IM [61]. Stern et al. conducted a prospective single-center study to evaluate the efficacy of isavuconazole for antifungal prophylaxis after allo-HSCT [112]. A total of 95 patients were included to receive a median of 90 days of oral/intravenous isavuconazole prophylaxis, among whom 7 discontinued prophylaxis due to toxicity and 3 due to breakthrough candidemia, but there was no case of BT-MCR. Overall, six patients (6.3%) died during the study, but no one was attributed to isavuconazole. A retrospective, single-center cohort study analyzing 98 patients with HMs receiving isavuconazole for prophylaxis also demonstrated good efficacy with only one case of BT-MCR [113].

Table 4 summarized the efficacy of posaconazole and isavuconazole for the prophylaxis of IM in patients with HMs. Based on information from Table 4, we can conclude that both posaconazole and isavuconazole play important roles in IM prophylaxis. However, there were still cases of BT-MCR during the prophylaxis of either of these two drugs, and most of these cases suffered from relapsed and/or refractory acute leukemia and prolonged neutropenia. In Table 4, 11 patients who were infected with BT-MCR during the prophylaxis of isavuconazole or posaconazole had a documented outcome, among whom 8 (72.7%) deceased, indicating a poor prognosis in this population.

## 7. Conclusions

The incidence rate of IM in HMs patients increased year by year, which became an important factor leading to the high mortality of this population [123]. In recent years, with the widespread use of new antifungal drugs, the mortality of IM in patients with HMs decreased, but it is still close to 50%, and BT-MCR occurs occasionally under the prophylaxis of *Mucorales*-active antifungals. The main form of IM in patients with HMs is PM, followed by ROCM and disseminated mucormycosis. Patients with PM have relatively specific imaging manifestations, such as “reverse halo” sign, multiple (≥10) nodules, and pleural effusion. L-AmB combined with surgical debridement is the most widely used first-line treatment of IM, and isavuconazole can also be used as an initial antifungal. Patients who are intolerant or refractory to the initial treatment of L-AmB can turn to isavuconazole or posaconazole for salvage treatment. Patients who are refractory to monotherapy or unable to tolerate the toxicities associated with high dose of L-AmB can consider combination treatment of antifungals. Iron chelating agents, deferiprone and deferasirox, granulocyte-stimulating factors or granulocyte macrophage colony-stimulating factor, interferon-γ, and hyperbaric oxygen can also be used as effective means of adjunctive therapy. With regard to antifungal prophylaxis, posaconazole or isavuconazole are recommended with low incidence of breakthrough infection. In general, the approaches to treating IM in patients with HMs are still limited, and early diagnosis, timely surgical debridement, and effective antifungal treatment are the most important ways to improve the survival rate of such patients.

## Figures and Tables

**Table 1:** Risk factors for IM in patients with HMs or HSCT.

	Characteristics of Studies			Risk Factors/Underlying Diseases, *n* (%)						
Reference	Time Period	Countries of Origin of Cases	Total Number of Patients	AL	Hyperglycemia	Neutropenia	Steroids	HSCT	GvHD	Voriconazole
Park et al., 2011 [7]	2001–2006	America	105	28 (26.7%)	46 (43.8%)	39 (37.1%)	59 (56.2%)	76 (72.4%)	61 (58.1%)	47 (44.8%)
Kontoyiannis et al., 2000 [16]	1989–1998	America	24	9 (37.5%)	6 (25.0%)	22 (91.7%)	20 (83.3%)	10 (41.7%)	2 (8.3%)	NA
Kontoyiannis et al., 2014 [10]	2004–2008	North America	74	NA	24 (32.4%)	45 (60.8%)	54 (73.0%)	32 (43.2%)	10 (13.5%)	40 (54.1%)
Lanternier et al., 2012 [13]	2005–2007	France	50	27 (54.0%)	9 (18.0%)	41 (82.0%)	13 (26.0%)	12 (24.0%)	5 (10.0%)	NA
Xhaard et al., 2012 [17]	2003–2008	France	29	12 (41.4%)	14 (48.3%),	6 (20.7%)	26 (89.7%)	29 (100%)	22 (75.9%)	12 (41.3%)
Pagano et al., 1997 [2]	1987–1995	Italy	37	32 (86.5%)	NA	33 (89.2%)	37 (100.0%)	NA	NA	NA
Muggeo et al., 2019 [18]	2009–2016	Italy	15	11 (73.3%)	5 (33.3%)	10 (66.6%)	13 (86.6%)	5 (33.3%)	NA	3 (20.0%)
Pagano et al., 2004 [19]	1987–2001	Multi-center	59	46 (78.0%)	10 (16.9%)	47 (79.7%)	59 (100.0%)	5 (8.5%)	NA	NA
Madney et al., 2019 [9]	2007–2017	Egypt	45	39 (86.7%)	NA	41 (91.1%)	16 (35.6%)	1 (2.2%)	1 (2.2%)	13 (28.9%)

AL = Acute Leukemia, HSCT = Hematopoietic stem cell transplantation, GvHD = Graft-versus-host disease. Some patients have multiple risk factors at the same time, and so, the total is more than 100%.

**Table 2:** Characteristics of patients with BT-MCR.

Reference	Number of Patients	Underlying Disease	BT-MCR Patients	Prophylactic Drugs	Characteristics of BT-MCR Patients
Rothe et al. (2021) [23]	15	AML, ALL, MDS, MM	6	Posaconazole (*n* = 5), isavuconazole *(n* = 1).	All patients required invasive mechanical ventilation and were treated with broad-spectrum antibiotics.
Lerolle et al. (2014) [24]	270	AML, GvHD	2	Posaconazole oral suspension.	Patients received broad spectrum antibiotics the month before BT-MCR onset, and were neutropenic at the time of BT-MCR onset.
Fontana et al. (2020) [21]	145	AML, MDS, HSCT	2	Isavuconazole.	Patients had a median duration of neutropenia of 25.5 days and relapsed/refractory acute leukemia.
Rausch et al. (2018) [25]	100	AML, ALL	4	Isavuconazole.	Patients were with prolonged neutropenia and relapsed/refractory leukemia at the time of BT-MCR.
Axell-House et al. (2021) [22]	103	Leukemia, MDS	103	*Mucorales*-active antifungals (9 cases of isavuconazole, 6 cases of posaconazole, 1 case of AmB); antifungals without anti-*Mucorales* activity (52 voriconazole, 22 echinocandins, 8 itraconazole, 5 echinocandin + voriconazole).	Patients developing BT-MCR while on *Mucorales*-active antifungals had a higher 42-day mortality (63% vs. 25%, *p* = 0.006).

BT-MCR, breakthrough mucormycosis; AML, acute myeloid leukemia; ALL, acute lymphoblastic leukemia; MM, multiple myeloma; MDS, myelodysplastic syndrome; HSCT, hematopoietic stem cell transplant; GvHD, graft-versus-host disease.

**Table 3:** Clinical and imaging characteristics of IM.

	Clinical Manifestations	Imaging Manifestations
Pulmonary mucormycosis	The triad of “cough, dyspnea, chest pain”, hemoptysis.	Exudation, cavity, ground-glass lesion, consolidation, pleural effusion, atelectasis, halo sign, reverse halo sign, air-crescent sign.
Rhino-orbital mucormycosis	Facial edema, pain, nasal congestion, rhinorrhea, eye pain, chemosis, proptosis, epiphora, and palatal ulcer destruction.	Thickened oedematous mucosa, opacification or obliteration of paranasal sinuses and bony destruction in CT, “black turbinate sign” in MRI.
Central nervous system mucormycosis	Headache, facial nerve palsy, ptosis, diplopia, hemiplegia, epilepsy.	Intraventricular “fungus balls”, thrombosis of intracranial arteries, the inflammatory alterations of the cavernous sinus and the involvement of adjacent structures (such as meninges).

**Table 4:** Prophylactic effect of posaconazole and isavuconazole on IM in patients with HMs.

Reference	Number of Patients	Underlying Disease	Drugs Used as Primary Prophylaxis	BT-MCR
Ullmann et al. [108]	301	GvHD	Posaconazole oral suspension	0
	299		Fluconazole	1
Cornely et al. [109]	304	AML, MDS	Posaconazole oral suspension	0
	298		Fluconazole or itraconazole	1
Pagano et al. [114]	260	AML	Posaconazole oral suspension	0
	241		Fluconazole or itraconazole	0
Cho et al. [115]	140	AML, MDS	Posaconazole oral suspension	2
	284		Fluconazole	Not described
Lerolle et al. [24]	270	AML, GvHD	Posaconazole oral suspension	2
Duarte et al. [110]	54	AML, MDS	Posaconazole tablets	0
Cornely et al. [111]	210	AML, MDS, GvHD	Posaconazole tablets	0
Chin et al. [116]	26	AML, MDS, ALL	Posaconazole tablets	3
Cornely et al. [117]	237	AML, MDS, GvHD	Intravenous posaconazole	0
Jeong et al. [118]	61	AML, ALL, GvHD	Intravenous posaconazole	0
Maertens et al. [119]	55	AML, MDS	Posaconazole intravenous formulation followed by oral suspension	0
Heimann et al. [120]	151	AML, ALL, MDS, lymphoma	Posaconazole tablets or intravenous formulation	0
Fontana et al. (2020) [21]	145	AML, MDS, GvHD	Isavuconazole	2
Rausch et al. [25]	100	AML, ALL	Isavuconazole	4
Stern et al. [112]	95	AL, MDS, lymphoma, GvHD	Isavuconazole	0
Cornely et al. [121]	24	AML	Isavuconazole	0
Rausch et al. [122]	140	AML	Posaconazole	0
	53		Isavuconazole	0
	84		Voriconazole	0
Bowen et al. [113]	98	AML, MDS, GvHD	Isavuconazole	1

BT-MCR, breakthrough mucormycosis; AL, acute leukemia; AML, acute myeloid leukemia; ALL, acute lymphoblastic leukemia; MDS, myelodysplastic syndrome; GvHD, graft-versus-host disease.

## Data Availability

Not applicable.

## Abbreviations

IM: invasive mucormycosis; HMs, hematological malignancies; CAM, COVID-19-associated mucormycosis; NAIMIs, non-Aspergillus invasive mold infections; allo-HSCT, allogeneic hematopoietic stem cell transplantation; IA, invasive aspergillosis; IC, invasive candidiasis; GvHD, graft-versus-host disease; BT-MCR, breakthrough mucormycosis; PM, pulmonary mucormycosis; ROCM, rhino-orbital-cerebral mucormycosis; L-AmB, liposome amphotericin B; CNS, central nervous system; MEC, minimum effective concentration; MIC, minimal inhibitory concentration; RdRp, RNA-dependent RNA polymerase; ELISA, enzyme-linked immunosorbent assay; LFIA, the lateral flow immunoassay; PCR/HRMA, PCR followed by high-resolution melt analysis; BAL, bronchoalveolar lavage; qPCR, quantitative PCR; NGS, next-generation sequencing; ALT, alanine aminotransferase; AST, aspartate aminotransferase.
